# In‐Situ Transmission Electron Microscope Investigation of the Calcination Behavior and Mechanism of Solid‐State Electrolyte Li_1_._3_Al_0_._3_Ti_1_._7_(PO_4_)_3_ (LATP)

**DOI:** 10.1002/smll.74761

**Published:** 2026-07-28

**Authors:** Chun‐Chia Chen, An‐Yuan Hou, Hua‐Jing Huang, Che‐Hung Wang, Chih‐Yen Lin, Yi‐Dong Lin, Yan‐Gu Lin, Chun‐Wei Huang, Jeng‐Kuei Chang, Wen‐Wei Wu

**Affiliations:** ^1^ Department of Materials Science and Engineering National Yang Ming Chiao Tung University Hsinchu Taiwan; ^2^ Scientific Research Division National Synchrotron Radiation Research Center Hsinchu Taiwan; ^3^ Department of Materials Science and Engineering Feng Chia University Taichung Taiwan; ^4^ Department of Chemical Engineering and R&D Center for Membrane Technology Chung Yuan Christian University Taoyuan Taiwan; ^5^ Center For the Intelligent Semiconductor Nano‐system Technology Research Hsinchu Taiwan

**Keywords:** atomic scale, in situ transmission electron microscopy, LATP, solid‐state electrolyte, synthesis process

## Abstract

Solid‐state electrolytes are key materials for all‐solid‐state lithium batteries (ASSLBs) due to their high safety, thermal stability, and ability to suppress lithium dendrite growth. Among them, Li_1_._3_Al_0_._3_Ti_1_._7_(PO_4_)_3_ (LATP), a NASICON‐type oxide electrolyte, is attractive for its high ionic conductivity and stability, but its formation mechanism during calcination remains poorly understood. Here, we combine ex situ XRD, Raman spectroscopy, and atomic‐scale in situ TEM to elucidate the structural evolution of LATP upon calcination. The results reveal a transformation from amorphous precursors to intermediate phases (Li_4_P_2_O_7_ and AlPO_4_), and finally to crystalline LATP at 800°C. In situ TEM directly visualized the structural evolution associated with Al incorporation into the LTP* lattice and the subsequent Ti‐site substitution, which reconstructs the phosphate framework and stabilizes the NASICON structure. High‐resolution TEM/FFT, supported by XPS and XAS analyses, confirms that Al substitution perturbs local Ti–O coordination while preserving global lattice stability. This study establishes, for the first time, the complete thermal‐induced formation mechanism of LATP at the atomic scale, providing new insights for optimizing synthesis and improving the performance of solid‐state electrolytes.

## Graphical abstract

This work reveals a resistive switching in high‐entropy oxide memristors through a coupling effect among oxygen vacancy dynamics, multi‐cation interactions, and lattice symmetry evolution. In situ TEM directly captures the monoclinic‐to‐cubic transformation and the formation of conductive pathways. These findings provide insight into the structure–redox–defect relationship and highlight the potential of high‐entropy oxides for next‐generation memory and neuromorphic applications.

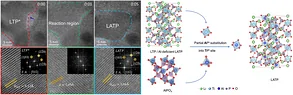

## 1 Introduction

Rechargeable lithium‐ion batteries (LIBs) have become the dominant energy storage technology for electric vehicles, smart grids, and portable electronics owing to their high energy density, long cycle life, and good reliability [1]. However, the conventional liquid electrolytes used in LIBs suffer from poor thermal stability and an inability to effectively suppress the growth of lithium dendrites [2, 3], which can cause short circuits, thermal runaway, and severe safety concerns [4, 5]. These intrinsic drawbacks fundamentally limit the further advancement of LIBs toward higher safety and energy density. Therefore, solid‐state electrolytes (SSEs) are regarded as a key enabler for next‐generation all‐solid‐state lithium batteries (ASSLBs). SSEs combine wide electrochemical stability windows and decent ionic conductivity with superior thermal and chemical stability compared to liquid electrolytes [6, 7, 8]. Among them, inorganic oxides are considered particularly promising due to their robustness against thermal and chemical degradation, although achieving sufficient densification often requires high sintering temperatures [9, 10].

Within oxide‐based SSEs, the NASICON (Na super ionic conductor) family has attracted considerable attention because of its open three‐dimensional framework and high structural symmetry, which facilitate fast lithium‐ion transport [7, 11, 12, 13, 14, 15, 16]. Owing to these advantages, NASICON‐type electrolytes have been extensively investigated for all‐solid‐state lithium batteries. Meesala et al. demonstrated the feasibility of employing NASICON‐type Li_1_._5_Al_0_._5_Ge_1_._5_(PO_4_)_3_ (LAGP) as a solid electrolyte in Li/LAGP/LiFePO_4_ all‐solid‐state batteries, showing stable cycling performance [17]. More recently, Paolella et al. developed a high‐performance NASICON‐based solid‐state lithium metal battery utilizing a thin LAGP electrolyte, an ultrathin lithium‐metal anode, and a high‐loading LiFePO_4_ cathode, highlighting the practical potential of NASICON electrolytes for high‐energy‐density solid‐state batteries [18]. In addition to bulk ionic conductivity, the electrochemical performance of NASICON electrolytes is also strongly influenced by interfacial phenomena. Paolella et al. further revealed that cation‐exchange reactions can occur at NASICON‐based electrolyte interfaces, highlighting the importance of interfacial chemistry in determining electrochemical behavior [19]. Furthermore, NASICON‐type LAGP electrolytes have been integrated with layered oxide cathodes to realize high‐performance solid‐state lithium metal batteries, demonstrating the practical applicability of NASICON electrolytes in high‐energy‐density battery systems [20]. Collectively, these studies highlight the technological significance of NASICON electrolytes and demonstrate the importance of both electrolyte properties and interfacial phenomena for achieving high‐performance solid‐state batteries. While germanium‐based NASICON electrolytes such as LAGP have demonstrated promising electrochemical performance in all‐solid‐state battery systems, titanium‐based phosphates have also attracted considerable attention owing to their favorable combination of structural stability, electrochemical performance, and processability. Among them, LiTi_2_(PO_4_)_3_ (LTP) is one of the most representative NASICON compounds and exhibits excellent electrochemical and thermal stability. However, its room‐temperature ionic conductivity remains limited (∼10^−^^6^ S cm^−^^1^) because of grain‐boundary resistance and restricted Li^+^ transport pathways [21, 22, 23]. To overcome this limitation, various aliovalent substitution strategies have been introduced to optimize the ionic conductivity of NASICON‐type phosphates. A wide range of dopants, including Zr^4^^+^, Nb^5^^+^, Ga^3^^+^, Mg^2^^+^, Si^4^^+^, and Sn^4^^+^, have been explored to tune the lattice structure and grain boundary properties, each exhibiting distinct effects on conductivity and structural stability [24, 25, 26]. Among these options, Al^3^^+^ substitution has been most extensively used [27, 28]. Due to its smaller ionic radius (0.535 Å vs. 0.605 Å for Ti^4^^+^), Al^3^^+^ effectively enhances lithium concentration and reduces grain boundary resistance. In addition, aluminum is earth‐abundant and cost‐effective compared with other potential dopants, which makes it particularly attractive for large‐scale synthesis. These advantages lead to the formulation of Li_1__+__x_Al_x_Ti_2__−__x_(PO_4_)_3_ (LATP), which achieves a significantly enhanced ionic conductivity of 10^−^^3^ S/cm [21, 29, 30, 31, 32].

Despite the promising properties of LATP, its synthesis and calcination remain challenging. Conventional solid‐state reactions often lead to the appearance of secondary phases such as Li_4_P_2_O_7_ and AlPO_4_, which are generally considered transient intermediates [33, 34, 35]. Li_4_P_2_O_7_ typically forms at moderate temperatures and gradually disappears as the NASICON framework develops. In contrast, AlPO_4_ is commonly regarded as a terminal and inert phase that impedes Li^+^ transport and limits densification; recent reports suggest that a small amount of AlPO_4_ may instead assist sintering, enhance densification, and thereby reduce grain boundary resistance [36, 37, 38]. These contrasting perspectives indicate that the role of AlPO_4_ in LATP remains controversial, motivating further investigation into its reaction behavior during sintering. Electron microscopy has also been employed to investigate dynamic processes in NASICON‐type solid electrolytes. Kaboli et al. employed in situ scanning electron microscopy to examine the thermal evolution of Li/LATP and Li/LAGP interfaces at elevated temperatures [39], whereas Camara et al. utilized in situ environmental SEM to investigate the sintering behavior and densification process of LATP under low‐pressure conditions [40]. These studies highlighted the capability of electron microscopy to capture interfacial and microstructural evolution in NASICON materials. However, direct observation of the phase formation pathway and crystallization process during LATP synthesis at the nanoscale remains scarce. Most studies on LATP formation still rely on ex situ techniques, such as XRD, Raman spectroscopy, and SEM, which provide only static snapshots of phase composition and morphology. Although in situ XRD and Raman spectroscopy have been applied to monitor phase transitions, these approaches provide only ensemble‐averaged structural information and lack the spatial resolution required to directly probe local reactions. As a result, the atomic‐scale dynamics of LATP formation, including nucleation, grain coalescence, and structural reorganization, remain unresolved. Given these challenges, in situ transmission electron microscopy (TEM) offers a powerful means to directly visualize the real‐time evolution of precursor particles under high‐temperature conditions. In this study, in situ TEM is combined with complementary techniques, including XRD, Raman spectroscopy, XPS, and XAS, to ensure reliable identification of structural and electronic changes. To the best of our knowledge, this is the first work to directly capture the atomic‐scale crystallization pathway of LATP during calcination. The findings not only provide mechanistic insights into LATP phase formation but also offer guidance for mitigating the formation of secondary phases during processing, thereby contributing to the optimization of LATP‐based solid electrolytes for practical applications.

## 2 Results and Discussion

**FIGURE 1:**
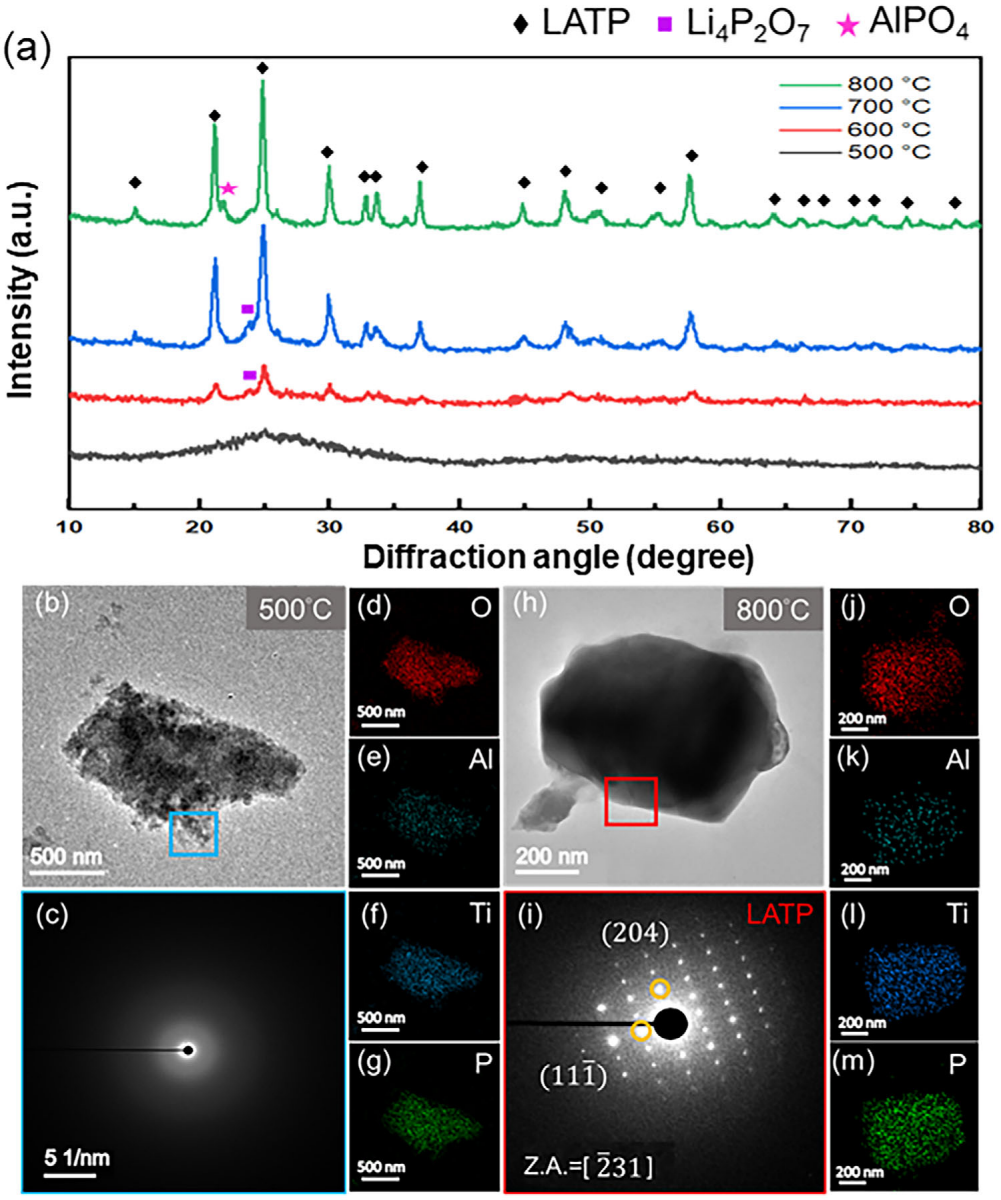
Structural and elemental characterization of LATP sintered at different temperatures. (a) XRD patterns show the progressive crystallization of LATP from amorphous (500°C) to highly crystalline (800°C), accompanied by transient Li_4_P_2_O_7_ and trace AlPO_4_ peaks. (b,c) STEM and SAED confirm the amorphous nature of the 500°C sample. (d–g) EDS mapping reveals homogeneous Al, Ti, P, and O distribution. (h,i) STEM and SAED at 800°C demonstrate well‐crystallized LATP with indexed diffraction spots. (j–m) EDS mapping at 800°C confirms uniform elemental incorporation without segregation.

The XRD patterns of the ex situ annealed samples are shown in Figure 1a. At 500°C, no distinct Bragg reflections were detected, indicating that the sample remained largely amorphous. Upon annealing at 600°C, diffraction peaks corresponding to the rhombohedral NASICON‐type phase of the R‐3c space group emerged, indexed to LiTi_2_(PO_4_)_3_ (JCPDS NO.35‐0754), which is isostructural with Li_1_._3_Al_0_._3_Ti_1_._7_(PO_4_)_3_ (LATP). The characteristic reflections were observed at 2θ = 21.21° and 24.90°, corresponding to the (104) and (113) planes of LATP, respectively. A minor peak at 23.79° was also detected and assigned to Li_4_P_2_O_7_, suggesting the coexistence of LATP with this intermediate phase. With further heating to 700°C, the diffraction peaks of LATP became sharper and more intense, while Li_4_P_2_O_7_ remained detectable. At 800°C, LATP exhibited well‐defined and highly crystalline reflections, with only a trace peak at 2θ ≈ 21.87° attributable to AlPO_4,_ which is typically formed as a secondary phase at elevated annealing temperatures [41, 42]. These findings confirm that LATP becomes the dominant crystalline phase at 800°C, with only minor traces of secondary phases remaining.

The scanning transmission electron microscopy (STEM) image of the 500°C pre‐annealed sample is shown in Figure 1b and reveals micrometer‐sized particles. The selected‐area electron diffraction (SAED) pattern is presented in Figure 1c, confirming its amorphous nature. The energy‐dispersive spectroscopy (EDS) elemental mapping in Figure 1d–g demonstrates a homogeneous distribution of Al, Ti, P, and O. After annealing at 800°C, the particles became more compact and interconnected, as displayed in Figure 1h. The SAED pattern in Figure 1i, taken along the [$\bar{2}31$] zone axis, shows sharp diffraction spots that can be indexed to the (204) and (111) planes of LATP. HRTEM observations thus confirm the high crystallinity of LATP at elevated temperatures. Elemental mapping in Figure 1j–m further confirms the homogeneous distribution of Al, Ti, P, and O without detectable segregation, indicating that Al^3^^+^ is uniformly incorporated into the LATP framework.

Ex situ XRD and TEM/EDS analyses revealed a progressive crystallization of LATP during annealing, accompanied by the transient formation of Li_4_P_2_O_7_ and AlPO_4_. At 800°C, the sample was predominantly composed of LATP, suggesting its thermodynamic stability at elevated temperatures. To further clarify the phase evolution and underlying reaction mechanism, in situ heating TEM was employed to directly monitor the real‐time structural dynamics of LATP at the atomic scale under high‐temperature conditions.

**FIGURE 2:**
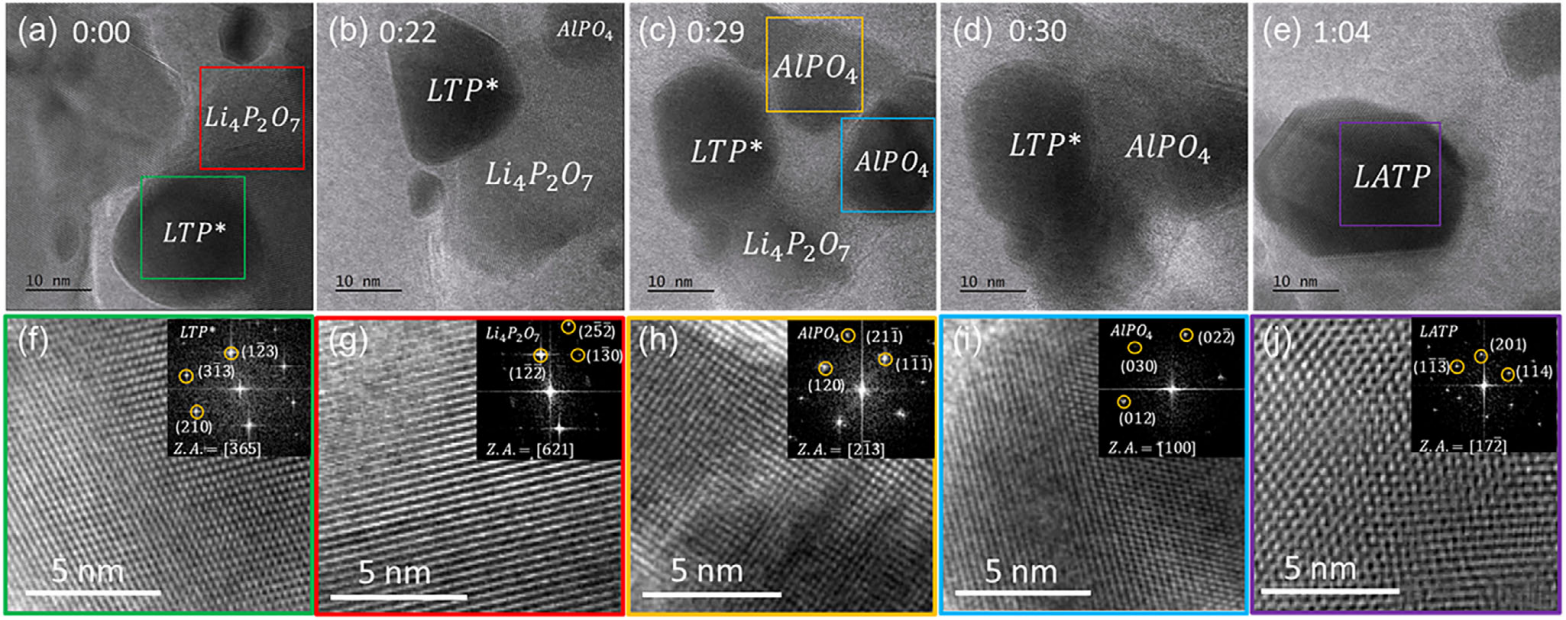
In situ TEM sequence of LATP formation at 800°C. (a–e) Real‐time TEM images reveal the evolution of LTP*, Li_4_P_2_O_7_, and AlPO_4_ during heating, accompanied by the gradual formation of LATP. (f–j) HRTEM and FFT analyses confirm phase identification and corresponding space groups at each stage.

The in situ TEM specimen corresponds to the same sample characterized by ex situ XRD at 500°C in Figure 1a. The absence of distinct Bragg reflections confirms that the material remains predominantly amorphous at this stage, ensuring consistency between the in situ heating experiments and the ex situ reference. An overview of the experimental procedure is provided in Figure S1. The specimen was then prepared from the pellet using FIB to obtain a lamella suitable for TEM observation, as shown in Figure S2. The heating parameters, presented in Figure S3, indicate that the sample was annealed stepwise from 500°C to 800°C, with the temperature increased by 50°C every 15 min and a ramp rate of 0.75°C s^−^^1^ between steps. The elemental distributions shown in Figure S4 indicate that O, Ti, P, Al, and Pt remain largely uniform during the intermediate annealing steps from 500°C to 780°C. Although pores developed on the specimen surface during heating, no distinct reactions were visible at low magnification, likely due to the small particle size associated with the sol–gel synthesis method. Clear crystallization and grain‐to‐grain reactions became more evident only at 800°C, as demonstrated in Figure 2, which provides the basis for the subsequent in situ TEM observations.

Figure 2 shows the sequential phase transformation of LATP precursors during in situ heating TEM at 800°C, with the calcination process provided in Movie S1. Initially, the particles were mainly identified as LiTi_2_(PO_4_)_3_ (LTP) or Al‐deficient LATP. For clarity, both of these Al‐deficient phases are hereafter referred to as LTP*, to distinguish them from the fully Al‐substituted LATP phase. In addition to LTP*, Li_4_P_2_O_7_ in the P12_1_/c1 space group was also observed, as shown in Figure 2a. With progressive heating, the interfacial contact between LTP* and Li_4_P_2_O_7_ increased, accompanied by local structural reorganization within the observed region, as displayed in Figure 2b. Subsequently, AlPO_4_ in the C222_1_ space group was detected in the vicinity of the LTP* and Li_4_P_2_O_7_ particles, as shown in Figure 2c. The AlPO_4_ domains expanded and coalesced into larger regions, thereby enlarging the interfacial area with LTP* and providing diffusion pathways essential for the subsequent transformation, as illustrated in Figure 2d. As the reaction advanced, LTP* and AlPO_4_ gradually converted into well‐crystallized LATP in the R‐3c space group, characterized by ordered lattice fringes and distinct phase boundaries, as shown in Figure 2e. The corresponding HRTEM images and FFT patterns in Figure 2f–j further validate the phase identification and confirm the space group assignments at each stage of the transformation. For clarity, the structural information and typical formation windows of these transient and final phases are summarized in Table S1, and the refined structural parameters of Li_1_._3_Al_0_._3_Ti_1_._7_(PO_4_)_3_ are provided in Table S2.

**FIGURE 3:**
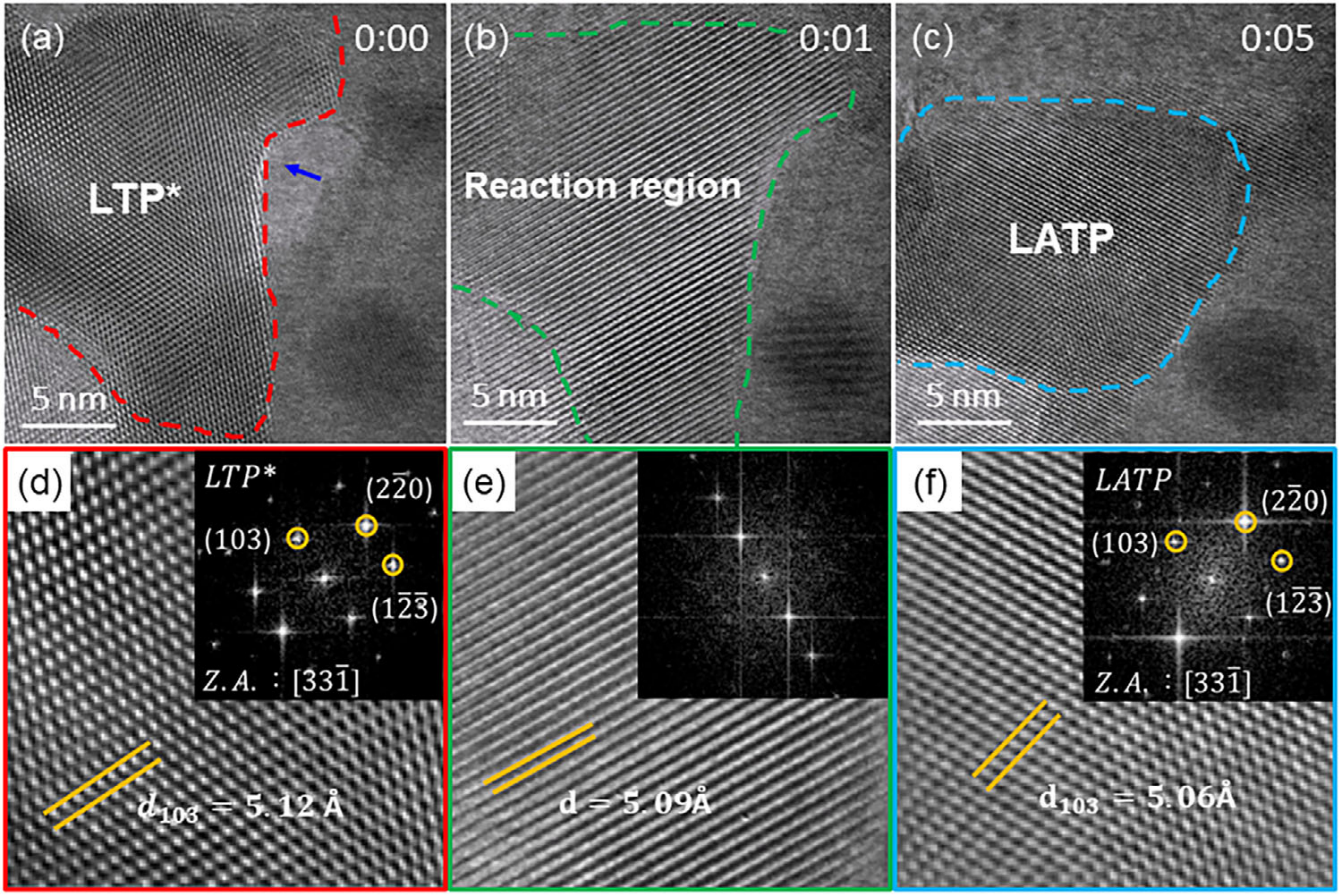
A series of HRTEM images and corresponding FFTs for the LATP forming process at 800°C. (a–c) The sequence of the grain reaction process for LTP* transform to LATP. (d–f) Corresponding HRTEM images and FFT are indicated in (a‐c), respectively.

To gain further insight into the crystallographic evolution at the atomic scale, HRTEM imaging was conducted under in situ heating conditions, while the calcination process is provided in Movie S2. Figure 3 presents a series of time‐resolved HRTEM images and corresponding FFT analyses, providing a magnified view of the transformation pathway, where the LTP* phase reacts with Al‐containing intermediate species to facilitate the formation of the final LATP structure at 800°C. In Figure 3a, the initial grain is indexed to LTP*, with lattice fringes corresponding to the (103) plane, giving an interplanar spacing of 5.12 Å. This confirms the structural identity of the precursor phase prior to reaction. As heating proceeds, subtle rearrangements of the lattice fringes can be observed (blue arrow), which may be associated with local interactions or rearrangements with the surrounding matrix, with the measured d‐spacing decreasing to 5.09 Å, as shown in Figure 3b. Such contraction indicates local lattice reorganization, which can be attributed to the substitution of Ti^4^^+^ by the smaller Al^3^^+^ cations [24, 25, 43], thereby stabilizing the NASICON‐type framework. This lattice contraction also implies a subtle modification of Li^+^ conduction pathways, which could influence ionic transport properties. Finally, Figure 3c reveals the formation of rhombohedral LATP, with the (103) reflection refined to 5.06 Å and well‐defined FFT spots indexed along the [33$\bar{1}$] zone axis. The gradual reduction in d‐spacing from 5.12 to 5.06 Å, together with the sharpening of FFT patterns, directly evidences the atomic‐scale structural reordering from LTP* to LATP, as shown in Figure 3d–f. Similar shrinkage of lattice spacing associated with enhanced crystallinity has also been reported in related NASICON‐type systems [44, 45]. Compared with the multi‐particle reaction pathway shown in Figure 2, the HRTEM sequence in Figure 3 emphasizes the local lattice evolution, thereby providing direct atomic‐scale confirmation of the phase evolution from LTP* toward the final LATP phase.

**FIGURE 4:**
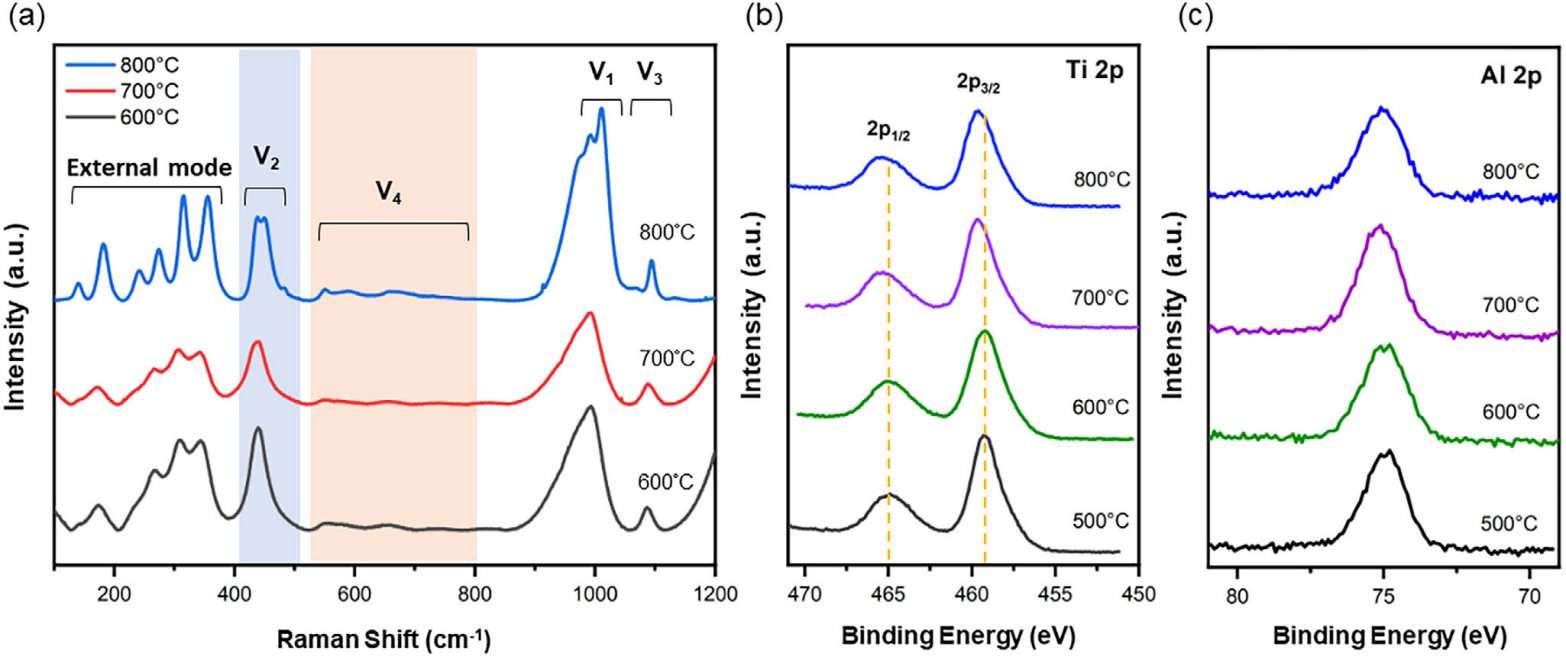
Raman and XPS analyses of LATP sintered at different temperatures. (a) Raman spectra of LATP sintered at temperatures ranging from 600°C to 800°C. (b) Ti 2p XPS spectra of LATP sintered at temperatures ranging from 500°C to 800°C. (c) Al 2p XPS spectra of LATP sintered at temperatures ranging from 500°C to 800°C.

To clarify the structural and chemical evolution of LATP during calcination, ex situ annealed samples were characterized by Raman and XPS spectroscopy. These spectroscopic techniques provide complementary insights into structural ordering, electronic states, and local coordination during annealing, and the results are further supported by TEM and XRD observations. As shown in Figure 4a, the Raman spectra of LATP annealed at 600°C–800°C exhibit the characteristic modes of the NASICON framework, including the external modes (200–500 cm^−^^1^), V_2_ (∼400–500 cm^−^^1^, Ti–O vibrations), V_4_ (500–800 cm^−^^1^, P–O bending), and V_1_ and V_3_ (800–1200 cm^−^^1^, P–O stretching). With increasing annealing temperature, peak intensities increase while the full width at half maximum (FWHM) decreases, indicating progressive atomic ordering and enhanced crystallinity of the NASICON structure.

Closer inspection highlights the 400–500 cm^−^^1^ band corresponding to Ti–O vibrations. The subtle variations in intensity observed in this region may originate from Al substitution for Ti, which induces local lattice distortion and modifies the framework stability. This behavior is consistent with similar spectral changes reported for Ni‐doped NASICON phases, as described by Zhou, Qiqi, et al. [46], where partial substitution also leads to lattice distortion and structural rearrangement. In addition, the 500–800 cm^−^^1^ region, corresponding to P–O bending modes, exhibits perturbations in peak position and shape. These features can be associated with the broadening of Raman modes and the appearance of additional bands, which have been attributed to the asymmetry caused by Al^3^^+^ substitution for Ti^4^^+^, as reported by Dai, Lijing, et al. [47]. Such substitution disrupts the original lattice symmetry and increases atomic disorder, leading to characteristic distortions in this spectral region. Notably, the relative changes in peak intensity with temperature imply that intermediate phases such as AlPO_4_ may already participate in the reaction process in the 600°C–700°C range. As shown in Figure 4b, the Ti 2p spectra of LATP samples annealed at 500°C–800°C display two main peaks at ∼459.0 eV (Ti 2p_3_⁄_2_) and ∼464.9 eV (Ti 2p_1_⁄_2_), with a spin–orbit splitting of ∼ 5.9 eV [48], characteristic of Ti^4^^+^ in an octahedral environment. No Ti^3^^+^ signal is observed, indicating that Ti remains in the +4 oxidation state across the studied temperatures. A slight shift of 0.3 eV toward higher binding energies is observed for both Ti 2p peaks with increasing annealing temperature, reflecting stronger electron binding and reduced electronic shielding. This trend is consistent with lattice distortion and local strain associated with aliovalent cation substitution. Considering the smaller ionic radius of Al^3^^+^ (0.535 Å) relative to Ti^4^^+^ (0.605 Å), these results support the possibility of partial Al incorporation at Ti sites, leading to electron density redistribution and the observed binding‐energy shift. Similar binding‐energy shifts of the Ti 2p core level upon trivalent cation substitution have also been reported in doped NASICON‐type phosphates, for example in Y^3^^+^‐doped LATP as noted by Wang, Ruoyu, et al. [49]. As shown in Figure 4c, the Al 2p spectra of LATP samples remain unchanged across the 500–800°C annealing range, indicating that the chemical environment of Al is stable. No significant peak shift or broadening is observed, suggesting that Al maintains its oxidation state and local coordination during annealing. Consistently, the P 2p and O 1s spectra presented in Figure S5 also show negligible variation with temperature, further confirming that the phosphate framework and oxygen environment remain stable during annealing. These results confirm that the binding‐energy shift observed in the Ti 2p spectra is associated with perturbation of the Ti sites rather than changes in the oxidation states of Al, P, or O.

**FIGURE 5:**
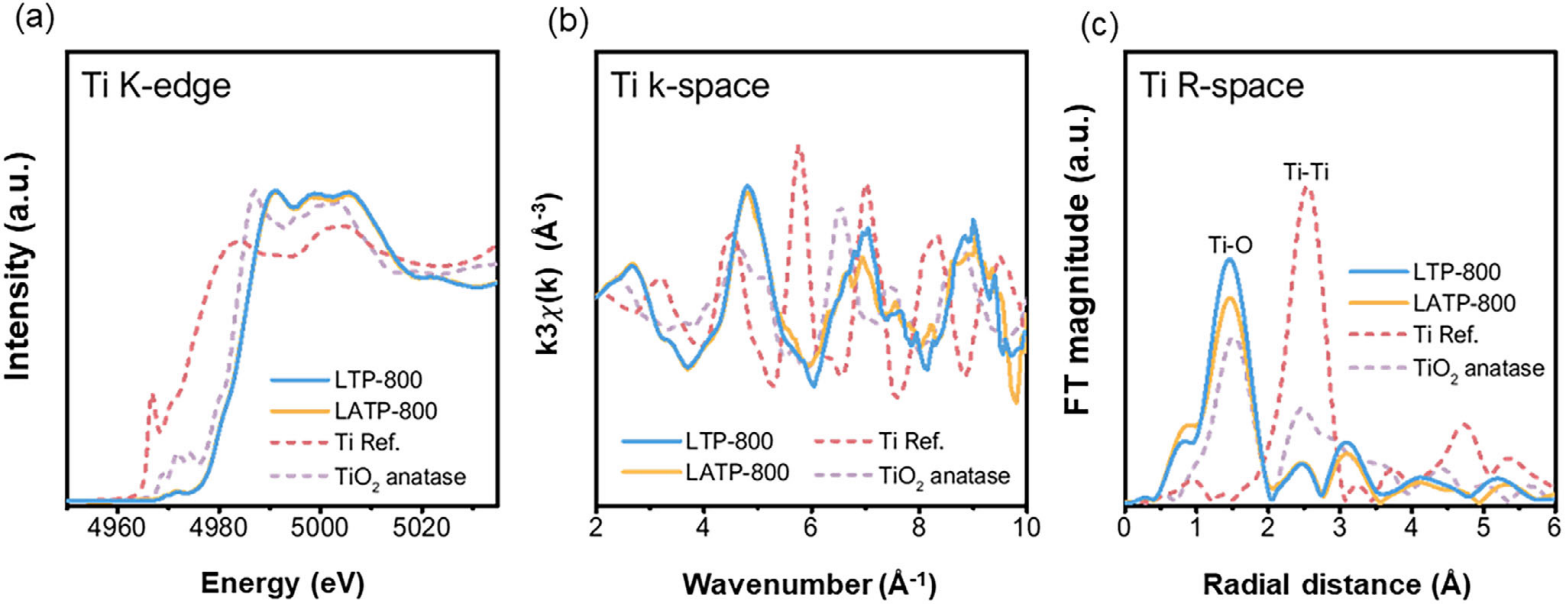
XANES and EXAFS analyses of the Ti local structure in LATP and LTP sintered at 800°C. (a) XANES spectra of the Ti K‐edge for LATP and LTP sintered at 800°C, (b) k3𝜒(k) EXAFS spectra for LATP and LTP sintered at 800°C, and (c) Magnitude of Fourier‐transformed k3 χ(k) spectra.

To further investigate the local structural effects of Al^3^^+^ substitution for Ti^4^^+^, Ti K‐edge x‐ray absorption spectroscopy (XAS) was performed. XAS provides element‐specific information with atomic‐scale resolution and can be divided into x‐ray Absorption Near Edge Structure (XANES) and Extended x‐ray Absorption Fine Structure (EXAFS) regions. XANES reveals the oxidation state and coordination symmetry of the absorbing atom, while EXAFS provides information on bond lengths, coordination numbers, and the degree of local structural order through interference of the photoelectron wave with neighboring atoms. In this study, a direct comparison was conducted between LATP and a separately synthesized LTP reference sample, in order to isolate and highlight the role of Al^3^^+^ substitution. This comparison clarifies how the incorporation of Al modifies the local electronic environment and structural configuration around Ti. As shown in Figure 5a, the XANES spectra of LATP‐800 and LTP‐800 exhibit a main absorption edge at ∼4984 eV with a weak pre‐edge feature at ∼4970 eV, characteristic of Ti^4^^+^ in octahedral coordination. The nearly identical edge positions indicate that Ti remains in the +4 oxidation state and that the overall coordination symmetry is largely preserved. The k‐space EXAFS χ(k^3^) oscillations are also similar, as shown in Figure 5b, suggesting comparable local atomic environments and bond‐length distributions, consistent with preserving the NASICON framework [50].

However, in the Fourier‐transformed R‐space spectra shown in Figure 5c, the first Ti–O coordination peak of LATP displays a markedly lower intensity than LTP, indicating a reduction in Ti coordination number. This observation can be attributed to local structural distortion and asymmetry induced by partial Al^3^^+^ substitution at Ti sites, providing further evidence of the effect of Al doping on the local electronic and structural environment around Ti. At higher R values (>2 Å), corresponding to Ti–P, Ti–Ti, or long‐range scattering paths, the peaks remain largely unchanged with no obvious phase shift, indicating that the overall bond lengths and long‐range framework connectivity are preserved despite local distortions.

Additional insights are provided by the Ti L‐edge and O K‐edge spectra shown in Figure S6. The Ti L‐edge spectra exhibit the characteristic four‐peak profile of Ti^4^^+^ in an octahedral crystal field, with the L_3_‐ and L_2_‐edges split into t_2g_ and e_g_ states. The nearly identical edge positions across different annealing temperatures confirm that Ti maintains the +4 oxidation state and that the overall NASICON framework is preserved. At the same time, the progressive sharpening of the spectral features with increasing temperature indicates enhanced crystal‐field splitting and reduced local disorder, suggesting that the Ti–O octahedra become increasingly ordered upon annealing. A similar trend is observed in the O K‐edge spectra, where the pre‐edge region exhibits two prominent peaks. These features are attributed to the transitions from O 1s to the hybridized O 2p–Ti 3d t_2g_ and e_g_ states, which are characteristic of Ti atoms in an octahedral coordination environment. The consistent positions of these t_2g_ and e_g_ peaks across all samples indicate that the local TiO_6_ geometry remains stable despite the different annealing temperatures. Furthermore, the features at higher energy, corresponding to O 2p–Ti 4sp hybridization, P–O–Ti linkages, and PO_4_^3^^−^ groups, remain at nearly the same positions. Their relative intensities evolve slightly with temperature, further corroborating the progressive ordering of the NASICON framework.

**FIGURE 6:**
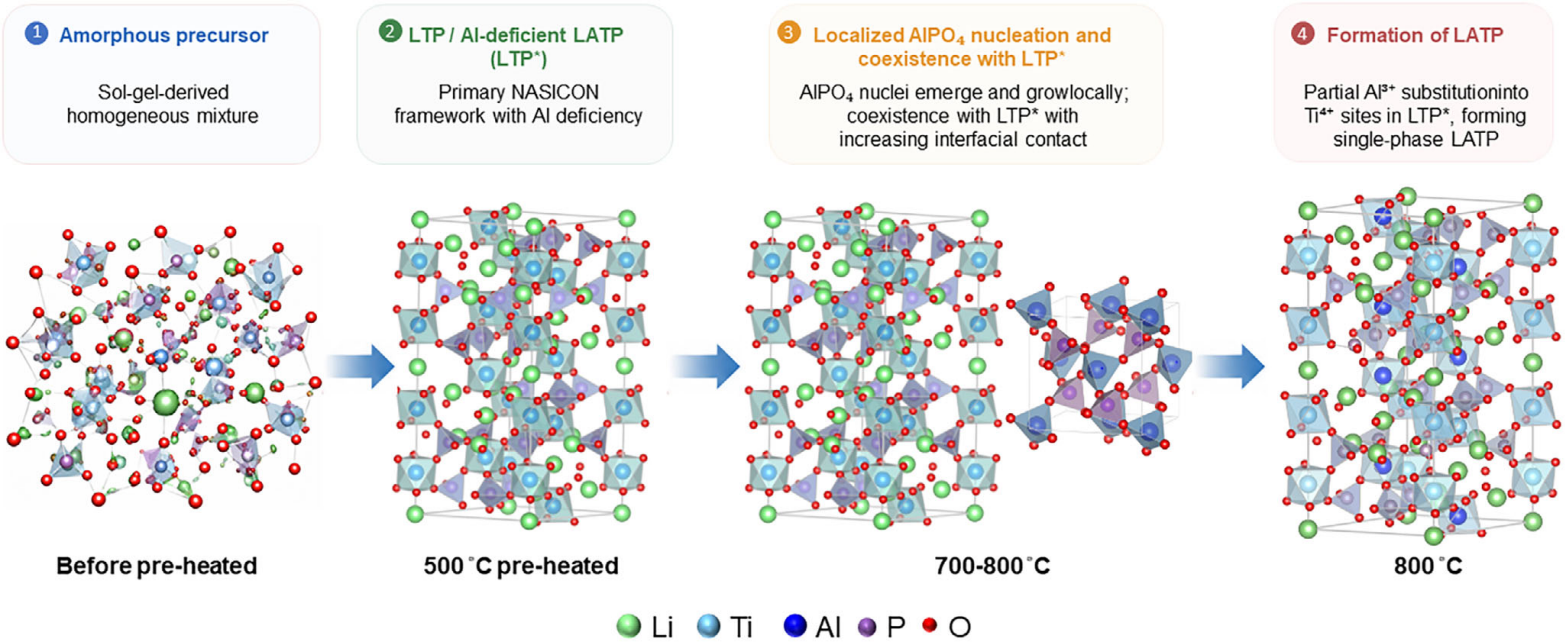
Schematic illustration of the phase evolution during LATP synthesis. The amorphous precursor evolves into LTP*, followed by the coexistence of LTP* and AlPO_4_. Al species derived from AlPO_4_ are progressively incorporated into Ti sites, leading to the formation of single‐phase LATP.

The proposed phase transformation pathway toward LATP formation is schematically illustrated in Figure 6. Starting from the sol–gel‐derived amorphous precursor, a primary LTP or Al‐deficient LATP framework (LTP*) is first formed. Upon further heating, localized AlPO_4_ nuclei emerge and coexist with LTP*, leading to increased interfacial contact and eventually the formation of single‐phase LATP through progressive Al incorporation. In the Li–Al–Ti–P–O system, LATP (Li_1_._3_Al_0_._3_Ti_1_._7_(PO_4_)_3_) is generally regarded as the desired end phase, and co‐sintering studies have shown that intermediate phases such as Li_4_P_2_O_7_ and AlPO_4_ often appear at moderate temperatures before gradually transforming into the NASICON framework. The phase evolution observed in this study is therefore consistent with the intermediate phases reported in the literature during LATP synthesis. In the present study, Li_4_P_2_O_7_ was identified as a minor transient phase during heating and gradually diminished as LATP formation progressed, whereas the appearance and growth of AlPO_4_ coincided with the subsequent formation of the NASICON‐type LATP structure. Additionally, the smaller ionic radius of Al^3^^+^ (0.535 Å) compared with Ti^4^^+^ (0.605 Å) enables Al^3^^+^ to effectively substitute into TiO_6_ octahedral sites, lowering the substitution energy barrier and thereby enhancing structural stability. Based on the in situ TEM observations, LATP formation is primarily associated with the interfacial reaction between LTP* and AlPO_4_, which becomes increasingly evident as AlPO_4_ develops and expands during heating. Notably, AlPO_4_ does not exist as a fully crystalline phase from the beginning; instead, it gradually forms as the temperature approaches 700–800°C. Al and P species are presumed to remain poorly ordered at lower temperatures, resulting in the absence of detectable diffraction features. Upon further heating, these components reorganize to crystallize as AlPO_4_, whose diffraction features then become detectable. During this process, Al^3^^+^ gradually diffuses into the LTP* lattice, substituting for Ti^4^^+^ and accompanied by lithium ions to maintain charge balance, ultimately stabilizing the NASICON‐type LATP structure. At low temperatures, Al^3^^+^ diffusion is relatively slow, so the reaction initiates preferentially at grain boundaries or particle interfaces and progressively extends into the grain interiors as temperature and time increase. In cases of insufficient Al^3^^+^ diffusion or incomplete reaction, residual Li_4_P_2_O_7_ or AlPO_4_ may be observed, reflecting local imbalances in phosphate and lithium stoichiometry. Consistent with this substitution mechanism, the HRTEM images in Figure 3 reveal that the interplanar spacing of the (103) reflection decreases progressively as LTP* transforms into LATP, providing direct atomic‐scale evidence for the structural reorganization induced by Al incorporation.

Collectively, the ex situ structural and chemical analyses, together with the HRTEM observations, suggest that LATP formation proceeds through the gradual substitution of Ti^4^^+^ by Al^3^^+^ in the LTP* lattice, accompanied by structural reorganization and lithium redistribution. These findings, consistent with Raman, XPS, and XAS results, support the conclusion that Al substitution modulates the coordination environment and structural ordering, ultimately leading to the stabilization of the NASICON framework. It is also worthwhile to consider whether the formation pathway observed in the present LATP system may be applicable to other NASICON‐type phosphate solid electrolytes, particularly LAGP. Previous studies have reported the presence of AlPO_4_ and Li_4_P_2_O_7_ during LAGP synthesis, and LAGP has been described as a solid solution of LiGe_2_(PO_4_)_3_ and AlPO_4_ [51]. These observations suggest similarities in the formation chemistry of LATP and LAGP. However, the currently available evidence is insufficient to conclude that LAGP follows the identical local phase‐evolution pathway observed in the present study. The in situ TEM approach demonstrated here may provide a useful platform for directly probing the crystallization behavior and reaction pathways of LAGP in future studies.

In addition to providing mechanistic insights into LATP formation, the practical applicability of the synthesized LATP was further evaluated in a solid‐state battery configuration. LATP was incorporated with a polymer matrix and lithium salt to form a composite solid electrolyte (CSE), and its electrochemical performance was examined using LiNi_0.8_Co_0.2_Mn_0.2_O_2_ (NCM‐811) as the cathode active material. As shown in Figure S7 and summarized in Table S3, the cell delivered a discharge capacity of 203 mAh g^−1^ at a current density of 25 mA g^−1^, demonstrating the potential of the synthesized LATP electrolyte for solid‐state battery applications.

## 3 Conclusions

In situ TEM was successfully employed to directly visualize the structural evolution and reaction pathways leading to the formation of LATP, offering atomic‐scale insights into its calcination behavior. By capturing the sequential evolution of LTP*, Li_4_P_2_O_7_, and AlPO_4_ during the formation of the thermodynamically stable NASICON phase, this study clarifies the role of Al substitution in modulating Ti coordination and driving structural reorganization. Notably, although AlPO_4_ is generally regarded as a terminal and unreactive phase, and has often been considered detrimental to ionic conductivity, our results suggest that AlPO_4_ remains involved in the phase evolution process under high‐temperature conditions. We attribute this unexpected reactivity to the small particle size of sol–gel‐derived powders, which enhances interfacial contact and lowers the kinetic barrier. This finding suggests that controlling precursor particle size not only improves reaction kinetics and phase purity, but also mitigates the negative impact of residual AlPO_4_ on ionic conductivity, thereby offering a practical pathway to optimize electrolyte performance. Collectively, these mechanistic insights provide valuable guidance for LATP fabrication, while the methodological framework demonstrated here underscores the broader potential of in situ TEM for studying dynamic processes in solid electrolytes, paving the way for the rational design of next‐generation NASICON‐type materials for all‐solid‐state batteries.

## 4 Experimental Section

### 4.1 Preparation of LATP Pellets

Li_1_._3_Al_0_._3_Ti_1_._7_(PO_4_)_3_ (LATP) powders were synthesized via a sol–gel method. Titanium isopropoxide, lithium nitrate, and aluminum nitrate were used as metal precursors, while ammonium dihydrogen phosphate served as the phosphorus source. Titanium isopropoxide was first hydrolyzed to form Ti(OH)_4_, which was subsequently dissolved in nitric acid to obtain a clear TiO_2_^+^ solution. Citric acid was then added as a chelating agent, followed by the addition of stoichiometric amounts of Li^+^, Al^3^^+^, and PO_4_^3^^−^ sources to form a homogeneous sol. The sol was dried at 80°C to obtain a gel. The obtained gel was subsequently pre‐heated in air at 500°C for 3 h to promote precursor decomposition and remove residual organic species derived from citric acid [52]. The resulting powders were then calcined in air at 800°C for 2 h to obtain crystalline LATP powders. After calcination, the powders were ground, pressed into pellets, and subjected to further characterization by x‐ray diffraction, Raman spectroscopy, x‐ray photoelectron spectroscopy, and x‐ray absorption spectroscopy.

### 4.2 Microstructural Characterization of Materials

X‐ray diffraction (XRD) was performed on powder samples using a Bruker D2 Phaser x‐ray Diffractometer with Cu Kα radiation (λ = 1.5418 Å) over a 2θ range of 10–80°. Raman spectra were collected from pelletized LATP samples using a HORIBA Raman spectrometer in the range of 100–1200 cm^−^^1^. X‐ray photoelectron spectroscopy (XPS) was conducted on thin pellet specimens with an ULVAC‐PHI, PHI Quantera II system, focusing on the surface chemical states. X‐ray absorption spectroscopy (XAS) was carried out on powder samples at the Taiwan Photon Source (TPS), National Synchrotron Radiation Research Center (NSRRC) using the quick‐scanning beamline.

### 4.3 In Situ TEM Observation

Pellets of sintered LATP powder were first pressed and subsequently gold‐coated before being thinned into electron‐transparent lamellae using a focused ion beam (FIB): ZEISS, Crossbeam 350. A protective platinum layer was first deposited on the cutting region to minimize surface damage during Ga ion milling. The prepared lamellae were further thinned to below 100 nm to ensure sufficient electron transparency for TEM imaging. After lift‐out, the specimens were transferred either onto conventional Cu grids for ex situ TEM analysis or onto Protochips heating chips for in situ TEM experiments. The heating chips, consisting of a micro‐heating element and a Si_3_N_4_ membrane window, enable rapid and stable localized heating inside the TEM while minimizing thermal drift, thus ensuring uniform heating conditions for dynamic observations. To minimize electron‐beam‐induced effects during the in situ TEM experiments, the electron beam was blanked throughout the heating process and was only illuminated during image acquisition. The influence of electron‐beam irradiation was separately evaluated by continuous beam exposure under identical imaging conditions, and the corresponding results are presented in Figure S8.

All in situ TEM movies and images were recorded using a field‐emission TEM (JEOL‐F200) at a 200‐kV accelerating voltage, and the elemental distribution was analyzed using EDS (Oxford EDS 100 TLE). During EDS analysis, the sample temperature was lowered to 400°C to prevent instrument malfunction.

### 4.4 Preparation of CSE

The IL was prepared by dissolving 1 m lithium bis(trifluoromethanesulfonyl)imide (LiTFSI, TCI, 98%) in *N*‐propyl‐*N*‐methylpyrrolidinium bis(fluorosulfonyl)imide (PMP‐FSI, Solvionic, 99.9%). In addition, 1 wt.% lithium difluoro(oxalato)borate (LiDFOB, Sigma–Aldrich, 99%) was used as an additive. Poly(vinylidene fluoride‐co‐hexafluoropropylene) (PVDF‐HFP, Mw: 400,000 g mol−1) and poly(methyl methacrylate) (PMMA, Mw: 50,000 g mol−1) were purchased from Sigma–Aldrich. Dimethylformamide (DMF, 99.8%) was obtained from J.T. Baker. The electrolyte slurry was prepared by mixing PVDF‐HFP, PMMA, LATP, and LiTFSI in a weight ratio of 12:3:10:75 in DMF. Subsequently, the electrolyte slurry was mixed with the IL in a mass ratio of 2:1. Polyethylene (PE, WSCOPE Corporation) membranes (thickness = 16 µm, porosity = 43%) were used as scaffolds and infiltrated with the electrolyte slurries. Finally, the CSE membranes were dried in a vacuum oven at 70°C for 24 h to completely remove the DMF solvent.

### 4.5 Preparation of NCM Cathodes

The NCM cathode slurry was prepared by mixing 85 wt.% NCM‐811 (UBIQ Technology Co., Ltd.), 5 wt.% Super P, 5 wt.% vapor‐grown carbon fibers (VGCF, Industrial Technology Research Institute, Taiwan), and 5 wt.% poly(vinylidenefluoride) (PVDF) binder in N‐methyl‐2‐pyrrolidinone (NMP, Alfa Aesar, 99%). The slurry was thoroughly stirred and then cast onto aluminum foil using a doctor blade. The coated electrodes were subsequently dried in a vacuum oven at 70°C for 12 h.

### 4.6 Material and Electrochemical Characterizations

For electrochemical testing, the electrodes were punched into discs corresponding to the CR2032 coin cell size. Lithium metal foil was used as the counter electrode. The coin cells were assembled in an argon‐filled glovebox (O_2_< 0.1 ppm, H_2_O < 0.1 ppm). Electrochemical performance tests were conducted at 30°C using a NEWARE CT‐4000 battery tester.

## Author Contributions

**Chun‐Chia Chen**: investigation, formal analysis, Writing – original draft, methodology. **Hua‐Jing Huang**: methodology, investigation. **Che‐Hung Wang**: methodology, investigation, formal analysis. **An‐Yuan Hou**: methodology, investigation, writing – original draft. **Yi‐Dong Lin**: data curation, formal analysis. **Yan‐Gu Lin**: resources, methodology, supervision. **Jeng‐Kuei Chang**: writing – review and editing, supervision, conceptualization, resources. **Chun‐Wei Huang**: writing – review and editing, formal analysis. **Wen‐Wei Wu**: conceptualization, funding acquisition, formal analysis, investigation, supervision, writing – review and editing. **Chih‐Yen Lin**: investigation, formal analysis.

## Conflicts of Interest

The authors declare no conflict of interest.

## Supporting information




**Supporting File 1**: smll74761‐sup‐0001‐SuppMat.docx.

**Supporting File 2**: smll74761‐sup‐0002‐MovieS1.mp4.

**Supporting File 3**: smll74761‐sup‐0003‐MovieS2.mp4.

## Data Availability

The data that support the findings of this study are available from the corresponding author upon reasonable request.
