# Supplementary material for: In‐Situ Transmission Electron Microscope Investigation of the Calcination Behavior and Mechanism of Solid‐State Electrolyte Li1.3Al0.3Ti1.7(PO4)3 (LATP)
Source: Small. 2026 Jul 28;22(53):e74761. doi: 10.1002/smll.74761 (PMC13592416; doi:10.1002/smll.74761)
Supplement: Supplementary file 1 [file SMLL-22-e74761-s001.docx]

Supporting Information

**In-situ Transmission Electron Microscope Investigation of the calcination behavior and mechanism of solid-state electrolyte Li₁.₃Al₀.₃Ti₁.₇(PO₄)₃ (LATP)**

*Chun-Chia Chen , An-Yuan Hou , Hua-Jing Huang , Che-Hung Wang , Chih-Yen Lin , Yi-Dong Lin , Yan-Gu Lin , Chun-Wei Huang , Jeng-Kuei Chang , and Wen-Wei Wu**

**List of Contents.**

Table S1 │**Crystal structures, space groups, and formation temperature ranges of Li₄P₂O₇, AlPO₄, and L(A)TP phases.**

Table S2 │**Structure parameter for Li_1.3_Al_0.3_Ti_1.7_(PO_4_)_3_.**

Table S3 │ **Reversible capacities of NCM-811 measured at various rates in the solid-state electrolyte cell.**

Fig. S1 │ **LATP synthesis and TEM sample preparation.**

Fig. S2 │ **Sample preparation of LATP by FIB for TEM investigation.**

Fig. S3 │ **Heating parameters.**

Fig. S4 │ ***In-situ* EDS mapping results.**

Fig. S5 │ **XPS spectra of LATP sintered at 500–800 °C. (a) P 2p, (b) O 1s.**

Fig. S6 │ **X-ray absorption near-edge structure (NEXAFS) spectra of LATP sintered at**

**500–800 °C.**

Fig. S7 │ **Galvanostatic charge–discharge curves of the NCM-811 || Li cell with**

**composite solid electrolyte.**

Fig. S8 │ **Evaluation of electron-beam-induced effects on the LATP specimen.**

Movie S1 │ ***In-situ* TEM video showing the grain reaction process of LATP at 800 °C.**

Movie S2 │ ***In-situ* HRTEM video showing the structural evolution of LATP during heating at 800 °C.**

**Table S1.** Crystal structures, space groups, and formation temperature ranges of Li₄P₂O₇, AlPO₄, and L(A)TP phases.

| Phase | Structure | Space group | Temperature (°C) |
| --- | --- | --- | --- |
| Li_4_P_2_O_7_ | **Monoclinic** | **P12₁/c1** | **650~700** |
| AlPO_4_ | **Orthorhombic** | **C2221** | **700~800** |
| L(A)TP | **Trigonal** | **R**$\bar{\boldsymbol{3}}$**c** | **500~800** |

**Table S2.** Structure parameter for Li_1.3_Al_0.3_Ti_1.7_(PO_4_)_3_.

| **Atom** | **Site** | **X** | **Y** | **Z** | **Occupancies** |
| --- | --- | --- | --- | --- | --- |
| **Li** | **6b** | **0.00000** | **0.00000** | **0.00000** | **1.00000** |
| **Li** | **18e** | **0.55000** | **0.00000** | **0.25000** | **0.06700** |
| **Ti** | **12c** | **0.00000** | **0.00000** | **0.14128** | **0.85000** |
| **Al** | **12c** | **0.00000** | **0.00000** | **0.14128** | **0.15000** |
| **P** | **18e** | **0.28715** | **0.00000** | **0.25000** | **1.00000** |
| **O** | **36f** | **0.17683** | **0.98516** | **0.19141** | **1.00000** |
| **O** | **36f** | **0.19006** | **0.16224** | **0.08272** | **1.00000** |

**a = 8.62273 Å	b = 8.62273 Å	c = 21.08164 Å**

**Unit-cell volume = 1357.453665 Å³**

**Table S3.** Reversible capacities of NCM-811 measured at various rates in the solid-state electrolyte cell.

| **Current density (mA g^-1^)** | **Reversible capacity (mAh g^-1^)** |
| --- | --- |
| **25** | **203** |
| **50** | **184** |
| **75** | **177** |
| **100** | **170** |
| **200** | **138** |
| **300** | **95** |

**Figure S1. LATP synthesis and TEM sample preparation.** (a) Preparing sol of LATP by adding precursors sequentially. (b) Drying the sol to obtain gel and pre-sintering under 500°C to obtain LATP powder. (c-d) Applying stress on the powder to get the LATP pellet. (e-f) Using Focus ion beam to prepare TEM specimen. (g-h) Transferring the specimen to TEM heating chip and its observation window.

**Figure S2. Sample preparation of LATP by FIB for TEM investigation.** (a) Pt deposition, (b) trench, (c-d) rough polish, (e) U-cut, (f-g) final polish and (h) cut.

**Figure S3.** **Heating parameters.** The sample was annealed in a stepwise manner from 500 °C to 800 °C, with the temperature increased by 50 °C every 15 minutes. The ramp rate between steps was 75 °C/s.

**Figure S4. *In-situ* EDS mapping results.** Initial LATP specimen was prepared for in situ TEM analysis by using focused ion beam (FIB). The EDS mapping results revealed a homogeneous elemental distribution of O, Ti, P, Al and Pt within the specimen from 25 to 780 °C during sintering process.

**Figure S5. XPS spectra of LATP sintered at 500–800 °C.** (a) P 2p, (b) O 1s.

**Figure S6. X-ray absorption near-edge structure (NEXAFS) spectra of LATP sintered at 500–800 °C.** (a) Ti L-edge, (b) O K-edge.

**Figure S7. Galvanostatic charge–discharge curves of the NCM-811 || Li cell with composite solid electrolyte.**

**Figure S8. Evaluation of electron-beam-induced effects on the LATP specimen.** (a–c) TEM image sequences acquired from three different regions of the LATP specimen after continuous electron-beam irradiation for 0, 20, 30, and 40 min under identical imaging conditions.

**Supplementary movies**

Movie S1: *In-situ* HRTEM observation of the progressive transformation of LTP* into LATP through reaction with AlPO₄ during heat treatment.

Movie S2: In-situ HRTEM observation of the LTP*–to–LATP transformation via grain coalescence during heat treatment.
